# Subnational inequalities in diphtheria–tetanus–pertussis immunization in 24 countries in the African Region

**DOI:** 10.2471/BLT.20.279232

**Published:** 2021-07-01

**Authors:** Katherine Kirkby, Nicole Bergen, Anne Schlotheuber, Samir V Sodha, M Carolina Danovaro-Holliday, Ahmad Reza Hosseinpoor

**Affiliations:** a Department of Data and Analytics, World Health Organization, 20 Avenue Appia, 1211, Geneva, Switzerland.; b Faculty of Health Sciences, University of Ottawa, Ottawa, Canada.; c Department of Immunization, Vaccines and Biologicals, World Health Organization, Geneva, Switzerland.

## Abstract

**Objective:**

To analyse subnational inequality in diphtheria–tetanus–pertussis (DTP) immunization dropout in 24 African countries using administrative data on receipt of the first and third vaccine doses (DTP1 and DTP3, respectively) collected by the Joint Reporting Process of the World Health Organization and the United Nations Children’s Fund.

**Methods:**

Districts in each country were grouped into quintiles according to the proportion of children who dropped out between DTP1 and DTP3 (i.e. the dropout rate). We used six summary measures to quantify inequalities in dropout rates between districts and compared rates with national dropout rates and DTP1 and DTP3 immunization coverage.

**Findings:**

The median dropout rate across countries was 2.4% in quintiles with the lowest rate and 14.6% in quintiles with the highest rate. In eight countries, the difference between the highest and lowest quintiles was 14.9 percentage points or more. In most countries, underperforming districts in the quintile with the highest rate tended to lag disproportionately behind the others. This divergence was not evident from looking only at national dropout rates. Countries with the largest inequalities in absolute subnational dropout rate tended to have lower estimated national DTP1 and DTP3 immunization coverage.

**Conclusion:**

There were marked inequalities in DTP immunization dropout rates between districts in most countries studied. Monitoring dropout at the subnational level could help guide immunization interventions that address inequalities in underserved areas, thereby improving overall DTP3 coverage. The quality of administrative data should be improved to ensure accurate and timely assessment of geographical inequalities in immunization.

## Résumé

**Objectif:**

Analyser les inégalités régionales en matière de taux d'abandon du vaccin diphtérique-tétanique-coquelucheux (DTC) dans 24 pays de la région Afrique, en se fondant sur les données administratives relatives à l'injection de la première et de la troisième dose du vaccin (DTC1 et DTC3, respectivement) collectées dans le cadre du Rapport conjoint de notification de l'Organisation mondiale de la Santé et du Fonds des Nations Unies pour l'enfance.

**Méthodes:**

Dans chaque pays, les districts ont été regroupés en quintiles selon le pourcentage d'enfants ayant interrompu la vaccination entre DTC1 et DTC3 (taux d'abandon). Nous avons employé six mesures synthétiques afin de quantifier les inégalités en matière de taux d'abandon entre districts, puis nous avons comparé ces taux avec les taux d'abandon nationaux ainsi qu’avec la couverture vaccinale DTC1 et DTC3.

**Résultats:**

Le taux d'abandon médian dans les différents pays s'élevait à 2,4% dans les quintiles affichant le taux le plus bas, et atteignait 14,6% dans les quintiles affichant le taux le plus haut. Dans huit pays, l'écart entre le quintile le plus haut et le quintile le plus bas était égal ou supérieur à 14,9 points de pourcentage. Dans la plupart des pays, les districts enregistrant les moins bonnes performances dans le quintile avec le taux le plus élevé étaient plus enclins à se laisser exagérément distancer par les autres. Une divergence qui passait inaperçue lorsqu'on ne tenait compte que des taux d'abandon nationaux. Les pays confrontés aux plus importantes inégalités en termes de taux d'abandon régional absolu étaient plus enclins, selon les estimations, à présenter une couverture vaccinale DTC1 et DTC3 moins élevée à l'échelle nationale.

**Conclusion:**

Dans la plupart des pays étudiés, nous avons constaté de fortes disparités entre districts concernant les taux d'abandon du vaccin DTC. Assurer un contrôle sur ces taux d'abandon au niveau régional pourrait orienter les campagnes de vaccination visant à réduire les inégalités dans les zones mal desservies, ce qui offrirait une meilleure couverture DTC3 globale. Enfin, la qualité des données administratives devrait être améliorée afin de fournir des évaluations précises et ponctuelles des variations géographiques en matière de vaccination.

## Resumen

**Objetivo:**

Analizar la desigualdad subnacional en el abandono de la inmunización contra la difteria, el tétanos y la tos ferina (DTP) en 24 países africanos utilizando datos administrativos sobre la recepción de la primera y la tercera dosis de la vacuna (DTP1 y DTP3, respectivamente) recogidos por el proceso de informes conjuntos de la Organización Mundial de la Salud y el Fondo de las Naciones Unidas para la Infancia.

**Métodos:**

Los distritos de cada país se agruparon en quintiles según la proporción de niños que abandonaron la vacunación entre la DTP1 y la DTP3 (es decir, la tasa de abandono). Se utilizaron seis medidas de resumen para cuantificar las desigualdades en las tasas de abandono entre los distritos y se compararon estas tasas con las tasas nacionales de abandono y la cobertura de inmunización DTP1 y DTP3.

**Resultados:**

La mediana de la tasa de abandono de la vacunación en todos los países fue del 2,4 % en los quintiles con la tasa más baja y del 14,6 % en los quintiles con la tasa más alta. En ocho países, la diferencia entre los quintiles más altos y más bajos era de 14,9 puntos porcentuales o más. En la mayoría de los países, los distritos con peores resultados en el quintil con la tasa más alta tendían a estar desproporcionadamente por detrás de los demás. Esta divergencia no era evidente si se observaban únicamente las tasas nacionales de abandono de la vacunación. Los países con las mayores desigualdades en la tasa absoluta de abandono de la vacunación subnacional tendían a tener una cobertura nacional estimada de inmunización DTP1 y DTP3 más baja.

**Conclusión:**

En la mayoría de los países estudiados hubo desigualdades claras en las tasas de abandono de la vacunación DTP entre los distritos. El seguimiento del abandono a nivel subnacional podría ayudar a orientar las intervenciones de inmunización que abordan las desigualdades en las zonas desatendidas, mejorando así la cobertura general de DTP3. La calidad de los datos administrativos debe mejorarse para garantizar una evaluación precisa y oportuna de las desigualdades geográficas en la inmunización.

## ملخص

**الغرض:**

لتحليل حالة التفاوت دون الوطنية في التسرب من التحصين ضد الدفتيريا والتيتانوس والسعال الديكي (DTP) في 24 دولة أفريقية باستخدام البيانات الإدارية عند تلقي جرعتي التحصين الأولى والثالثة (DTP1 وDTP3 على التوالي)، والتي تم جمعها بواسطة عملية الإبلاغ المشتركة لمنظمة الصحة العالمية، ومنظمة الأمم المتحدة للطفولة.

**الطريقة:**

تم تجميع المناطق في كل دولة في مجموعات خمسية وفقًا لنسبة الأطفال الذين تسربوا بين الجرعتين DTP1 وDTP3 (أي معدل التسرب). استخدمنا ستة مقاييس موجزة لقياس حالات التفاوت في معدلات التسرب بين المناطق، وقمنا بمقارنة هذه المعدلات مع معدلات التسرب الوطنية وتغطية التحصين ضد الجرعتين DTP1 وDTP3.

**النتائج:**

كان متوسط معدل التسرب عبر الدول 2.4% في المجموعات الخمسية ذات المعدل الأقل، و14.6% في المجموعات الخمسية ذات المعدل الأعلى. في ثماني دول، كان الفرق بين أعلى وأقل شريحتين 14.9 نقطة مئوية أو أكثر. في معظم الدول، كانت المناطق ذات الأداء الضعيف في المجموعة الخمسية ذات أعلى معدل، تميل إلى التدهور بشكل غير متناسب عن غيرها من المناطق. لم يكن هذا الاختلاف واضحًا من مجرد النظر فقط إلى معدلات التسرب الوطنية. تميل الدول التي لديها أكبر تفاوتات في معدل التسرب المطلق دون الوطني، إلى أن يكون لديها تغطية تحصين وطنية تقديرية أقل من الجرعتين DTP1 وDTP3.

**الاستنتاج:**

كانت هناك تفاوتات ملحوظة في معدلات التسرب من التحصين ضد الدفتيريا والتيتانوس والسعال الديكي بين المناطق في معظم الدول التي شملتها الدراسة. يمكن لمراقبة التسرب على المستوى دون الوطني أن تساعد في توجيه تدخلات التحصين التي تعالج التفاوتات في المناطق المحرومة، وبالتالي تحسين التغطية الشاملة للجرعة DTP3. يجب تحسين جودة البيانات الإدارية لضمان التقييم الدقيق وفي الوقت المناسب للتفاوتات في التحصين.

## 摘要

**目的:**

使用世界卫生组织和联合国儿童基金会联合报告中收集的有关接种第一剂和第三剂疫苗（分别为 DTP1 和 DTP3）的管理数据，分析24个非洲国家关于白喉-破伤风-百日咳 (DTP) 免疫接种的地区不均问题。

**方法:**

根据要接种 DTP1 和 DTP3 却未完成全部接种的儿童比例（即辍接种率），将每个国家的地区按五分位置划分。我们使用六种综合测量方法来量化地区之间辍接种率的不均衡，并将其与国家辍接种率以及 DTP1 和 DTP3 免疫覆盖率进行比较。

**结果:**

各国的辍接种率中位数最低的五分之一为 2.4%，最高的五分之一为 14.6%。在八个国家中，最高和最低五分之一之间的差距为 14.9 个百分点或更多。在大多数国家，辍接种率最高的五分之一中，表现不佳的地区，远远落后于其他地区。仅从国家间的辍接种率来看，这种差异并不明显。地区辍接种率不均衡程度最大的国家，往往具有较低的预计国家 DTP1 和 DTP3 免疫覆盖率。

**结论:**

在大多数受调查的国家中，各地区之间 DTP 辍接种率存在明显不均。监测地区辍接种率，有助于对服务欠缺地区的不均衡问题进行免疫干预指导，从而提高 DTP3 的整体覆盖率。应提高管理数据的质量，以确保准确、及时地评估免疫接种方面的地域不均问题。

## Резюме

**Цель:**

По коэффициентам выбытия проанализировать неравенство на субнациональном уровне в области охвата иммунизацией коклюшно-дифтерийно-столбнячной вакциной (КДС) в 24 африканских странах с использованием административных данных о получении первой и третьей доз вакцины (КДС-1 и КДС-3 соответственно), собранных в рамках Единого процесса отчетности Всемирной организации здравоохранения и Детского фонда Организации Объединенных Наций.

**Методы:**

Районы в каждой стране были сгруппированы по квинтилям согласно доле детей, выбывших между получением доз КДС-1 и КДС-3 (т. е. коэффициент выбытия). Мы использовали шесть сводных показателей для количественной оценки неравенства по коэффициентам выбытия между районами и сравнили коэффициенты с национальными коэффициентами выбытия и охватом иммунизацией КДС-1 и КДС-3.

**Результаты:**

Для всех стран в квинтилях с наиболее низкими коэффициентами выбытия медианное значение выбытия составило 2,4%, в квинтилях с наиболее высокими коэффициентами выбытия — 14,6%. В восьми странах разница в показателях между квинтилями с наиболее высокими и наиболее низкими коэффициентами выбытия составила 14,9 процентного пункта или больше.. В большинстве стран отстающие районы в квинтилях с наиболее высокими коэффициентами выбытия имели тенденцию к непропорциональному отставанию по сравнению с другими районами. Это расхождение не было очевидным, если ориентироваться только на национальные коэффициенты выбытия. Страны с самым высоким уровнем неравенства по абсолютным коэффициентам выбытия на субнациональном уровне, как правило, имели более низкий оценочный уровень национального охвата иммунизацией КДС-1 и КДС-3.

**Вывод:**

В большинстве изученных стран наблюдалось неравенство между районами в коэффициентах выбытия в период получения КДС-вакцины. Мониторинг выбытия на субнациональном уровне может помочь в проведении мероприятий по иммунизации, направленных на устранение неравенства в недостаточно обслуживаемых районах, тем самым улучшая общий охват иммунизацией КДС-3. Качество административных данных должно быть улучшено для обеспечения точной и своевременной оценки географического неравенства в области охвата иммунизацией.

## Introduction

Routine childhood immunization reduces morbidity and mortality and is associated with a wide host of benefits for child development, health and the economy. Moreover, universal immunization is the focus of major global health and development initiatives, including the Global Vaccine Action Plan and the Immunization Agenda 2030 endorsed by the World Health Assembly in 2020.^1^^,^^2^ Universal immunization is also implicit in the third sustainable development goal,^3^ which is to ensure healthy lives and promote well-being for all at all ages. In practice, coverage with three doses of the combined diphtheria, tetanus and pertussis (DTP) vaccine is commonly used as a performance indicator for routine vaccine delivery because it is included in the Expanded Programme on Immunization in all countries and it involves several doses.^4^ In addition, the proportion of children immunized with the first DTP vaccine dose (DTP1) who failed to get the third dose (DTP3), hereafter referred to as the DTP1–DTP3 dropout rate, provides an indication of: (i) the immunization system’s potential to reach all children and retain them within a basic vaccination series; (ii) service utilization and quality; and (iii) the minimum level of continuity of care achievable. The dropout rate is effectively the proportion of children who did not finish the vaccination course.

National estimates of routine immunization coverage can conceal large inequalities in coverage or access to vaccines within a country.^5^^–^^11^ Consequently, geographical monitoring of immunization coverage was emphasized in the Global Vaccine Action Plan, which specified a target of 80% DTP3 coverage for each district in addition to attaining 90% coverage nationally.^12^ Similarly, the Immunization Agenda 2030 set the ambitious equity goal of ensuring, “everyone is protected by full immunization, regardless of location, age, socioeconomic status or gender-related barriers.”^1^^,^^13^ Although demographic, socioeconomic and cultural factors have all been reported to influence immunization coverage in African countries,^14^^–^^17^ monitoring subnational inequalities has practical advantages.^18^ First, location-specific strategies for improvement can be developed. Second, benchmarks can be established from multicountry comparisons.^10^

Data sources, data quality and data analysis are important considerations in assessing immunization coverage at the subnational level.^19^ Administrative data (i.e. data routinely collected by health-care systems) are well suited for monitoring immunization coverage at the district level because they often include timely information about all vaccines administered and data can be collected cost-effectively. However, the availability and accuracy of the data depend on the reliability of the underlying reporting system.^20^^,^^21^ In addition, the value of the denominator (i.e. the target population size) for coverage estimates must be accurate.^22^ Another technical consideration for multicountry comparisons of district-level inequality is that countries do not all have the same number of comparison groups (e.g. districts) and it is, therefore, difficult to accurately compare levels of inequality – this is a so-called resolution issue.^18^^,^^23^

The aim of this study was to calculate the DTP1–DTP3 dropout rate in each district in 24 countries in the African Region, from the reported number of DTP1 and DTP3 vaccine doses administered. The method we used addresses the resolution issue by grouping districts in a country into quintiles and circumvents the problem of estimating the population denominator because the dropout rate is based on the number of vaccine doses administered. Consequently, our estimates are more likely to be accurate. However, immunization dropout excludes children who did not receive any vaccine doses.

## Methods

We collected administrative data from 2018 on subnational coverage of DTP1 and DTP3 in districts in countries from the African Region through the Joint Reporting Process of the World Health Organization (WHO) and the United Nations Children’s Fund (UNICEF).^24^ Data on national coverage were based on WHO/UNICEF national immunization coverage estimates for 2018,^25^ which use reported administrative data as well as data from surveys, publications and the grey literature.^26^ National DTP1–DTP3 dropout rates were derived from country-reported administrative data on immunization coverage.

**Fig. 1:**
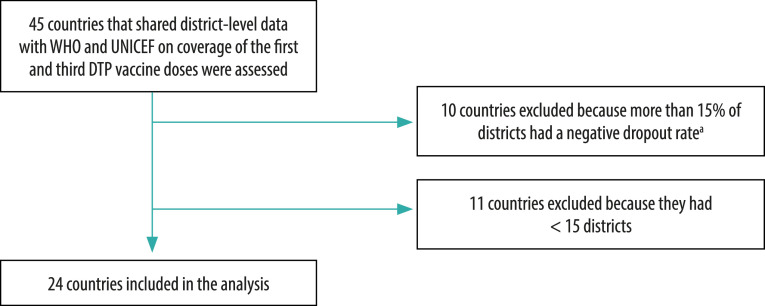
Country selection, analysis of subnational inequality in diphtheria–tetanus–pertussis immunization dropout rate, African Region, 2018

DTP: diphtheria, tetanus and pertussis; UNICEF: United Nations Children’s Fund; WHO: World Health Organization.

^a^ The dropout rate was negative if reported coverage of the first vaccine dose was less than coverage of the third dose. The DTP1–DTP3 dropout rate was defined as the proportion of children immunized with the first diphtheria, tetanus and pertussis vaccine dose (DTP1) who failed to get the third dose (DTP3).

In our analysis, we express the DTP1–DTP3 dropout rate as a percentage, which was calculated as the difference between the number of third and first doses administered divided by the number of first doses × 100. If the DTP1–DTP3 dropout rate was negative (which indicates that more third than first doses were reported and that there were possible data quality issues) in a particular district, that district was excluded from the analysis. Of 45 African countries for which district data were available on DTP1 and DTP3, 10 were excluded because more than 15% of districts had a negative dropout rate and, consequently, the remaining districts may not have accurately represented inequality in the country. A further 11 countries were excluded because they had fewer than 15 districts – 15 was the minimum required to evaluate subnational inequality as districts had to be divided into five quintiles each containing at least three districts. The cut-off threshold for the percentage of districts with a negative dropout rate (i.e. 10, 15 or 20%) was determined using a sensitivity analysis that took into account its effect: (i) on the number of countries finally included in the study (the larger the number, the more generalizable the results); and (ii) on median dropout rates across the countries included (the only aggregate figures in the analysis). Details of the results of the sensitivity analysis are available from the data repository.^27^ Fig. 1 shows the selection of study countries and Table 1 provides details of the 24 countries included in the analysis.

**Table 1:** Country characteristics, analysis of subnational inequality in diphtheria–tetanus–pertussis immunization dropout rate,^a^ African Region, 2018^b^

Country	No. districts with DTP immunization data reported^c^	District population, median (IQR)	No. districts included in analysis (% of districts with data reported)	No. districts excluded from analysis (% of districts with data reported)^d^
Angola	170	3 185 (1 338–6 447)	153 (90)	17 (10)
Benin	77	3 920 (2 934–4 917)	72 (94)	5 (6)
Botswana	24	1 946 (1 108–3 232)	22 (92)	2 (8)
Burkina Faso	70	10 012 (7 765–14 492)	64 (91)	6 (9)
Burundi	46	8 123 (6 736–9 611)	46 (100)	0 (0)
Cameroon	189	3 444 (1 679–6 153)	175 (93)	14 (7)
Central African Republic	35	4 170 (3 286–5 392)	33 (94)	2 (6)
Chad	117	4 966 (3 101–7 931)	115 (98)	2 (2)
Côte d’Ivoire	83	9 619 (5 731–14 293)	71 (86)	12 (14)
Democratic Republic of the Congo	519	6 166 (4 640–9 040)	507 (98)	12 (2)
Ethiopia	852	3 152 (1 739–4 680)	782 (92)	70 (8)
Guinea	38	10 815 (6 632–13 316)	38 (100)	0 (0)
Kenya	47	32 241 (18 481–41 426)	42 (89)	5 (11)
Madagascar	114	7 061 (4 945–10 057)	107 (94)	7 (6)
Malawi	28	21 437 (12 381–32 741)	26 (93)	2 (7)
Mali	75	10 232 (4 445–14 144)	74 (99)	1 (1)
Mauritania	55	2 376 (1 147–3 524)	50 (91)	5 (9)
Mozambique	161	5 310 (2 868–8 017)	141 (88)	20 (12)
Niger	72	11 077 (5 293–18 801)	71 (99)	1 (1)
Nigeria	772	8 448 (6 490–11 431)	745 (97)	27 (3)
South Sudan	78	4 930 (3 019–7 766)	70 (90)	8 (10)
Togo	41	6 073 (3 934–9 446)	36 (88)	5 (12)
Uganda	122	10 883 (7 895–16 073)	108 (89)	14 (11)
Zimbabwe	63	5 204 (3 566–9 405)	56 (89)	7 (11)

DTP: diphtheria, tetanus and pertussis; IQR: interquartile range.

^a^ Children were defined as dropping out of diphtheria–tetanus–pertussis immunization if they received the first vaccine dose but not the third.

^b^ Administrative data for 2018 on coverage of the first and third diphtheria, tetanus and pertussis vaccine doses in districts were collected through the Joint Reporting Process of the World Health Organization and the United Nations Children’s Fund.^24^

^c^ The number of districts for which data were reported by a country may not equal the total number of districts in the country.

^d^ Districts were excluded from the analysis if more third than first vaccine doses were reported, which indicates possible data quality issues.

For each country, districts were divided into quintiles according to their DTP1–DTP3 dropout rate. This approach makes it possible: (i) to assess within-country inequalities; and (ii) to compare and benchmark inequalities between countries, without the results being biased by outliers or by changes in the number of districts in a country within a given year.^18^ Quintile 5 contains the 20% of districts with the lowest dropout rates and quintile 1 contains the 20% with the highest rates. The dropout rate for each quintile was calculated by averaging the dropout rates of the districts included, weighted by the denominator (i.e. the number of first doses) for the district. Details of the number of first and third vaccine doses administered and the dropout rate in each quintile for the 24 study countries are available from the data repository.^28^

Six summary measures of inequality in dropout rates between districts were calculated: (i) the absolute difference; (ii) the relative difference; (iii) the weighted mean difference from the mean; (iv) the weighted index of disparity; (v) the population attributable risk; and (vi) the population attributable fraction.^29^^–^^31^ Details of their calculation are shown in Table 2. Absolute and relative differences are simple measures of inequality that express differences between the highest and lowest district quintiles within each country. Complex measures of inequality (i.e. the weighted mean difference from the mean and its relative version, the weighted index of disparity) were also calculated to indicate the magnitude of the difference between each district quintile and the national average – these measures consider the population size in each quintile. For ease of interpretation, only the simple measures of inequality are reported in the results when they showed similar patterns to complex measures.

**Table 2:** Summary measures of inequality in diphtheria–tetanus–pertussis immunization dropout rate

Summary measure	Measure type	Description^a,b^	Formula	Interpretation
Absolute difference	Simple measure of absolute inequality	The difference between the indicator value for quintile 1 (γ*_high_*), with the highest dropout rate, and the value for quintile 5 (γ*_low_*), with the lowest dropout rate		A high absolute value indicates a high level of inequality (range: 0 to 100 percentage points)
Relative difference	Simple measure of relative inequality	The difference between the indicator value for quintile 1 (γ*_high_*), with the highest dropout rate, and the value for quintile 5 (γ*_low_*), with the lowest dropout rate, divided by the value for quintile 1 (γ*_high_*)		The relative difference is zero if there is no difference between the highest and lowest quintiles and it is one when the difference is at its maximum (range: 0 to 1)
Weighted mean difference from the mean	Complex measure of absolute inequality	The weighted average of the difference between the indicator value for quintile *j* (γ*_j_*), and the national average (*μ*), Differences are weighted by each quintile’s share of the total population (*p_j_*),		The mean difference from the mean is zero if there is no inequality between quintiles; larger values indicate higher levels of inequality
Weighted index of disparity	Complex measure of relative inequality	The weighted average of the difference between the indicator value for quintile *j* (γ*_j_*) and the national average (*μ*) divided by the national average (*μ*) and multiplied by 100. Differences are weighted by each quintile’s share of the total population (*p_j_*)		The index of disparity is zero if there is no inequality between quintiles; larger values indicate higher levels of inequality
Population attributable risk	Complex measure of absolute inequality	The difference between the indicator value for the reference quintile with the best performance for the indicator (γ*_ref_*) and the national average (*μ*)		The larger the population attributable risk, the higher the level of inequality between quintiles; the population attributable risk is zero if no further improvement can be achieved
Population attributable fraction	Complex measure of relative inequality	The population attributable risk divided by the national average (*μ*) and multiplied by 100		The larger the population attributable fraction, the higher the level of inequality between quintiles; when the population attributable fraction is zero, there is no difference between the national average and the best-performing quintile

^a^ The dropout rate was defined as the proportion of children immunized with the first diphtheria, tetanus and pertussis vaccine dose (DTP1) who failed to get the third dose (DTP3).

^b^ For each country, districts were divided into quintiles according to dropout rate.

In addition, the population attributable risk and its relative version, the population attributable fraction, were calculated to quantify the improvement in national dropout rates that could be achieved if subnational inequality were reduced or eliminated within a country; that is, respectively, (i) if the dropout rate in the quintiles with a rate greater than the national average became equal to the national average; or (ii) if the national average equalled the dropout rate in quintile 5. The impact on national DTP3 coverage of reducing or eliminating subnational inequality was estimated using administrative data on DTP1 and DTP3 coverage collected through the WHO/UNICEF Joint Reporting Process rather than WHO/UNICEF national immunization coverage estimates, which could have yielded erroneous results for some countries because different data sources would have been used for subnational and national rates.

## Results

**Fig. 2:**
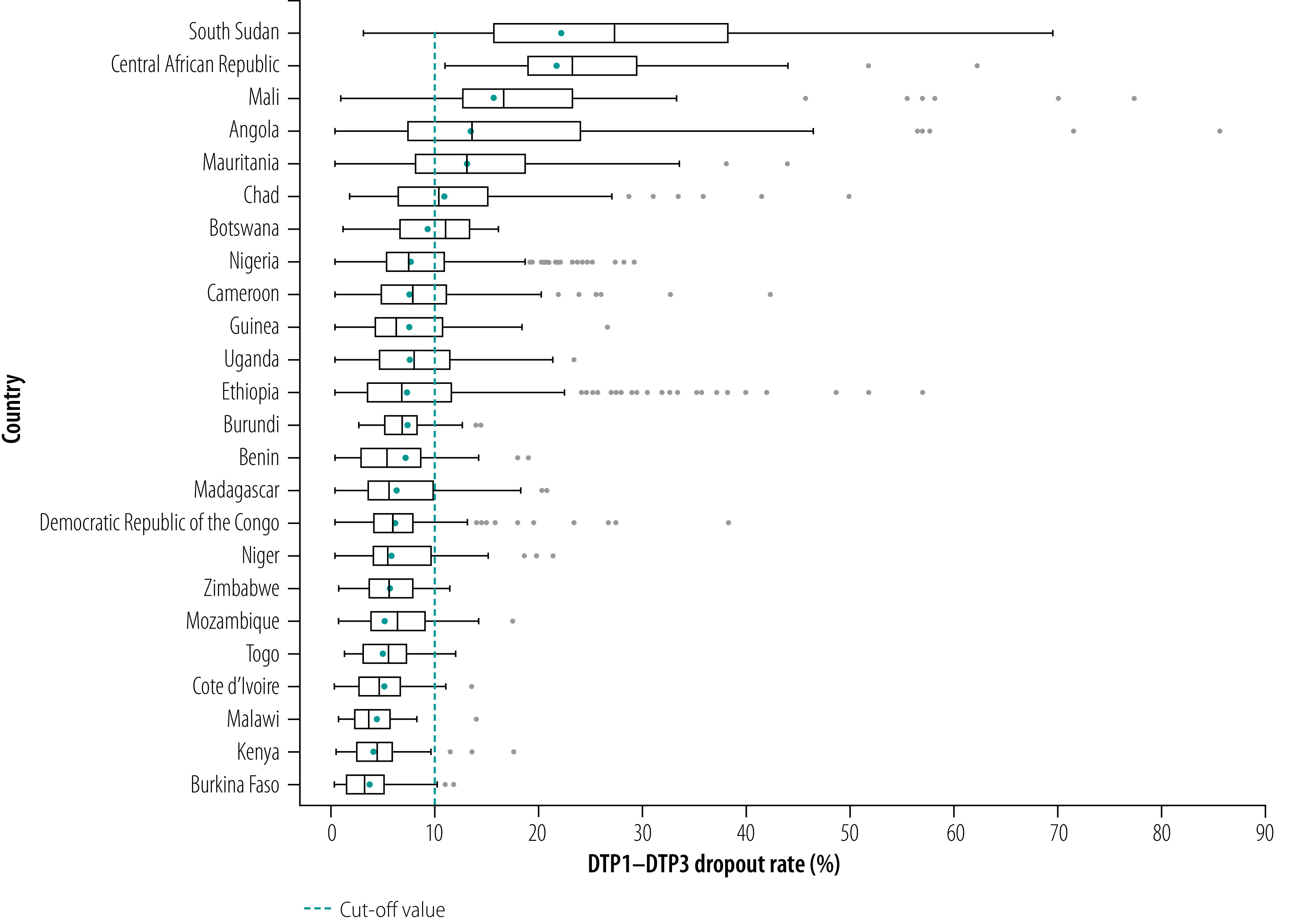
Diphtheria–tetanus–pertussis immunization dropout rate, by country and district, African Region, 2018

DTP1: first dose of diphtheria, tetanus and pertussis vaccine; DTP3: third dose of diphtheria, tetanus and pertussis vaccine.

Notes: The DTP1–DTP3 dropout rate was defined as the proportion of children immunized with the first diphtheria, tetanus and pertussis vaccine dose (DTP1) who failed to get the third dose (DTP3). District dropout rates for 2018 in each country were derived from data on vaccine coverage collected through the Joint Reporting Process of the World Health Organization and the United Nations Children’s Fund. The blue dots indicate average national DTP1–DTP3 dropout rates. Each box plot displays the distribution of the reported DTP1–DTP3 dropout rates among districts in each country: (i) the centre line indicates the median; (ii) the box represents the interquartile range (i.e. the middle 50% of values); (iii) the whiskers cover 1.5 times the interquartile range (i.e. all data points within 1.5 times the width of the adjoining box); and (iv) the grey dots indicate districts that are outliers beyond 1.5 times the interquartile range. The cut-off value of 10% dropout rate is commonly used.

According to WHO/UNICEF national immunization coverage estimates for 2018, national DTP1 coverage across the 24 countries ranged from 55% in Chad to 98% in Botswana (median: 88%; 95% confidence interval, CI: 82–94) and national DTP3 coverage ranged from 41% in Chad to 95% in Botswana (median: 80%; 95% CI: 72–88). The national average DTP1–DTP3 dropout rate derived from WHO/UNICEF Joint Reporting Process administrative data ranged from 3.5% in Burkina Faso to 22.6% in South Sudan (median: 7.3%; 95% CI: 5.6–8.3). However, national dropout rates mask considerable subnational variation (Fig. 2). For instance, in Ethiopia the national average DTP1–DTP3 dropout rate in 2018 was 7.3%, but between the lowest and highest districts the rates ranged from 0% to 57%. Of the 24 countries, six had a national average dropout rate above 10% and, in 12, a quarter of districts exceeded this threshold. Details of the results for all summary measures are shown in Table 3.

**Table 3:** Subnational inequality in diphtheria–tetanus–pertussis immunization dropout rate,^a^ by summary measure, African Region, 2018

Country^b^	Summary measure of subnational inequality in diphtheria–tetanus–pertussis immunization dropout rate^c^					
	Absolute difference,% points	Relative difference	Weighted mean difference from the mean, % points	Weighted index of disparity, %	Population attributable risk, % points	Population attributable fraction, %
Angola	32.3	0.90	7.1	48.4	10.8	74.1
Benin	11.2	0.90	3.5	49.8	5.7	81.7
Botswana	10.8	0.72	2.5	22.1	7.1	62.8
Burkina Faso	6.9	0.92	2.1	55.2	3.3	85.2
Burundi	7.1	0.65	2.0	27.0	3.4	47.2
Cameroon	14.9	0.90	3.6	45.9	6.3	78.8
Central African Republic	29.8	0.70	5.7	25.4	9.4	42.0
Chad	17.7	0.79	4.5	41.1	6.4	58.4
Côte d'Ivoire	8.7	0.90	2.4	48.6	4.0	80.3
Democratic Republic of the Congo	8.6	0.77	2.3	37.3	3.6	58.7
Ethiopia	18.3	0.91	4.8	62.1	6.0	77.1
Guinea	13.6	0.83	4.3	55.3	5.1	65.2
Kenya	9.8	0.88	2.2	48.4	3.1	70.6
Madagascar	12.5	0.88	3.4	56.9	4.4	72.6
Malawi	6.2	0.82	1.8	42.2	2.8	67.6
Mali	21.9	0.72	4.5	27.3	8.0	48.7
Mauritania	23.9	0.87	6.1	43.8	10.2	73.3
Mozambique	10.0	0.82	2.8	41.5	4.6	68.2
Niger	11.6	0.84	2.2	39.8	3.5	61.7
Nigeria	12.9	0.80	3.6	42.4	5.3	63.0
South Sudan	40.6	0.83	10.7	43.9	16.0	65.8
Togo	7.2	0.79	2.0	34.6	3.9	67.2
Uganda	13.3	0.84	3.8	45.2	5.8	69.3
Zimbabwe	8.4	0.83	2.1	33.3	4.6	73.4

^a^ Children were defined as dropping out of diphtheria–tetanus–pertussis immunization if they received the first vaccine dose but not the third.

^b^ Data on district dropout rates for 2018 in each country were derived from data on vaccine coverage collected through the Joint Reporting Process of the World Health Organization and the United Nations Children’s Fund.

^c^ Definitions of the summary measures and details of how they were calculated are shown in Table 2.

**Fig. 3:**
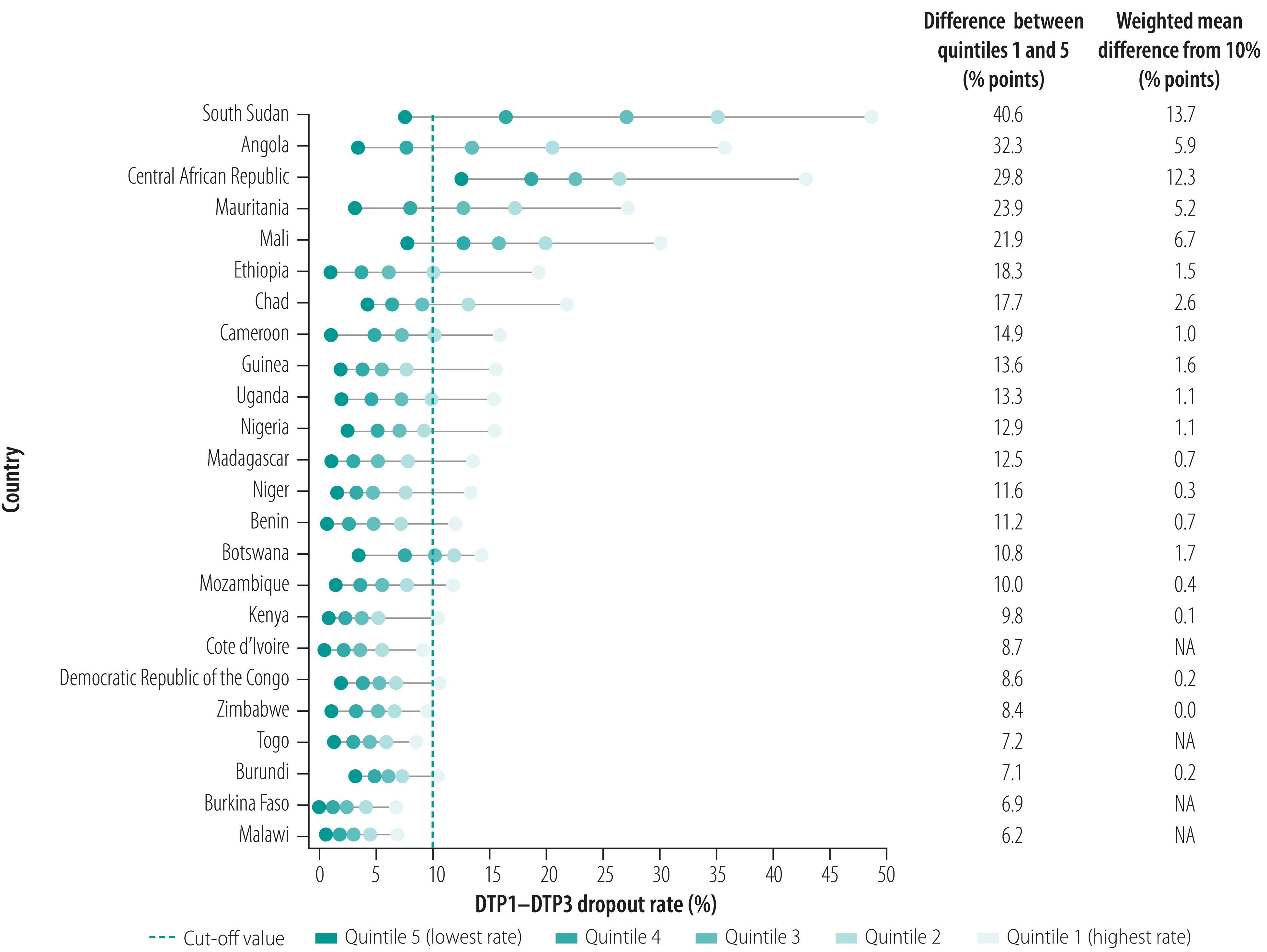
Diphtheria–tetanus–pertussis immunization dropout rate, by country and district quintile, African Region, 2018

DTP1: first dose of diphtheria, tetanus and pertussis vaccine; DTP3: third dose of diphtheria, tetanus and pertussis vaccine; NA: not applicable.

Notes: The DTP1–DTP3 dropout rate was defined as the proportion of children immunized with the first diphtheria, tetanus and pertussis vaccine dose (DTP1) who failed to get the third dose (DTP3). District dropout rates for 2018 in each country were derived from data on vaccine coverage collected through the Joint Reporting Process of the World Health Organization and the United Nations Children’s Fund. Districts in each country were divided into quintiles according to their DTP1–DTP3 dropout rate, with quintile 5 containing the 20% of districts with the lowest rates and quintile 1 containing the 20% with the highest rates. The dots represent weighted average values for each quintile. The values for the weighted mean difference from 10% are not listed for Burkina Faso, Côte d’Ivoire, Malawi or Togo because no quintile in these countries had a dropout rate greater than 10%.

Across study countries, the median dropout rate for quintile 5 was 2.4% (95% CI: 1.7–3.7) compared with a median of 14.6% (95% CI: 11.1–17.8) for quintile 1. Five of the 24 countries – Angola, the Central African Republic, Mali, Mauritania and South Sudan – had large subnational inequality (i.e. the difference between quintiles 1 and 5 was 20 percentage points or more) and the weighted mean difference from the 10% threshold was more than 5 percentage points (Fig. 3). Subnational inequality was also relatively high in Cameroon, Chad and Ethiopia (i.e. the difference between quintiles 1 and 5 was 14.9 to 18.3 percentage points), but the weighted mean difference from the 10% threshold was only 1.0 to 2.6 percentage points. In most countries, there was a substantial gap in dropout rate between quintile 1 and other quintiles, which indicated that there was a group of underperforming districts that lagged disproportionately behind the rest in routine immunization dropout rates. In 19 of the 24 countries, the weighted average dropout rate in quintile 1 was above 10%. Although dropout rate estimates can be spuriously low in districts with a small population, on average the populations of districts in quintile 1 were no smaller than those of districts in other quintiles.

**Fig. 4:**
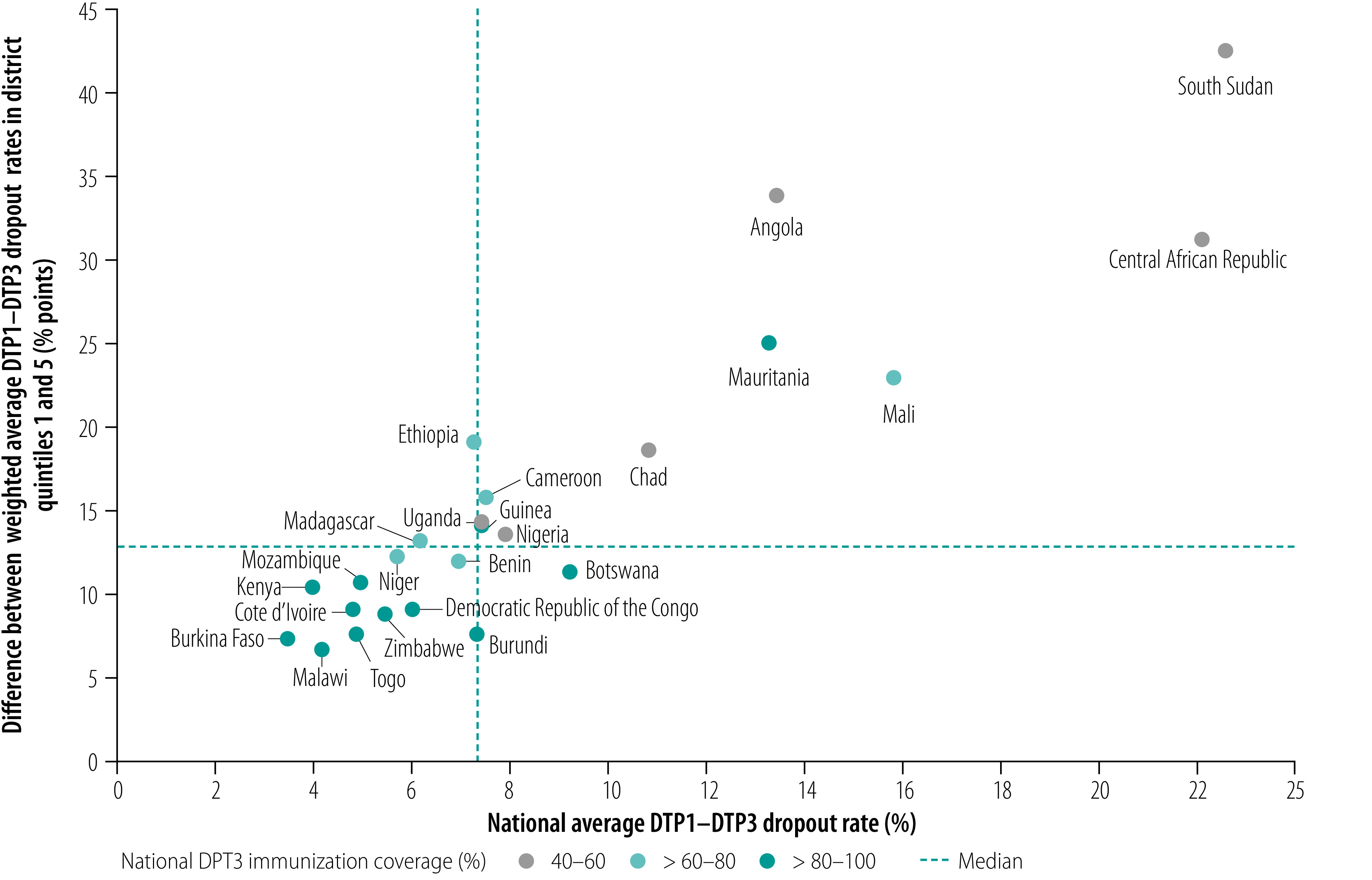
Subnational inequality in diphtheria–tetanus–pertussis immunization dropout rate versus national dropout rate, by country, African Region, 2018

DTP1: first dose of diphtheria, tetanus and pertussis vaccine; DTP3: third dose of diphtheria, tetanus and pertussis vaccine.

Notes: The DTP1–DTP3 dropout rate was defined as the proportion of children immunized with the first diphtheria, tetanus and pertussis vaccine dose (DTP1) who failed to get the third dose (DTP3). District and national average dropout rates for 2018 in each country were derived from data on vaccine coverage collected through the Joint Reporting Process of the World Health Organization (WHO) and the United Nations Children’s Fund (UNICEF). Districts in each country were divided into quintiles according to their DTP1–DTP3 dropout rate, with quintile 5 containing the 20% of districts with the lowest rates and quintile 1 containing the 20% with the highest rates. The difference between weighted average rates in quintiles 1 and 5 represents subnational inequality. National DTP3 immunization coverage rates were based on WHO/UNICEF national immunization coverage estimates.

The absolute difference in dropout rate between quintiles 1 and 5 in a country positively correlated with the national dropout rate, such that subnational inequality tended to increase as the national average dropout rate increased (Fig. 4). Angola, the Central African Republic, Mali, Mauritania and South Sudan had the highest national dropout rates among the study countries and the largest differences between quintiles 1 and 5. In addition, Angola, the Central African Republic and South Sudan had low DTP3 coverage, according to WHO/UNICEF national immunization coverage estimates (Fig. 4). Eight countries had both lower absolute subnational inequality (i.e. the difference between quintiles 1 and 5 was less than 10 percentage points) and low national average DTP1–DTP3 dropout rates (i.e. below 6 percentage points): Burkina Faso, Côte d’Ivoire, the Democratic Republic of the Congo, Kenya, Malawi, Mozambique, Togo and Zimbabwe. These countries all also had estimated DTP3 coverage rates above 80% (Fig. 4).

Although subnational inequality in the dropout rate generally increased as the national dropout rate increased, the situation varied from country to country. For example, Burundi and Ethiopia both had national dropout rates of 7.3% but subnational inequality was over two times higher in Ethiopia than Burundi: the difference between quintiles 1 and 5 was 18.3 percentage points and 7.1 percentage points in the two countries, respectively. In other countries, subnational inequality was similar despite varying national averages. For instance, in Botswana and Kenya, the difference between quintiles 1 and 5 was 10.8 and 9.8 percentage points, respectively, despite national average dropout rates differing by more than a factor of two: 9.2% and 4.0% in the two countries, respectively. Fig. 3 shows that districts in quintile 1 in Kenya fell substantially behind other districts, whereas quintiles 2 to 5 were clustered together around a relatively low average dropout rate, which meant the national average was low. On the other hand, in Botswana average dropout rates in the quintiles were distributed more evenly and hence the national average was higher.

**Fig. 5:**
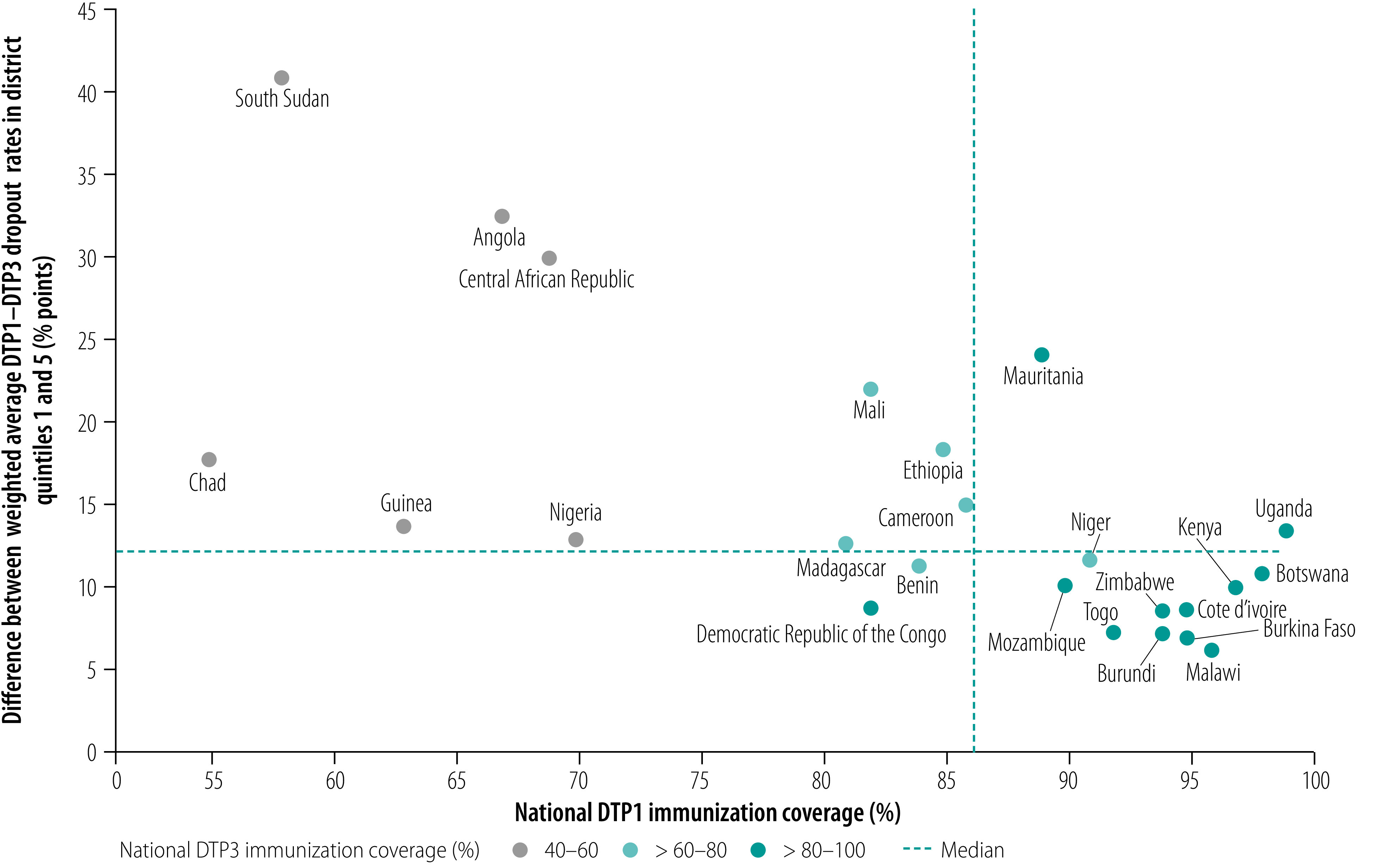
Subnational inequality in diphtheria–tetanus–pertussis immunization dropout rate versus national coverage of the first vaccine dose, by country, African Region, 2018

DTP1: first dose of diphtheria, tetanus and pertussis vaccine; DTP3: third dose of diphtheria, tetanus and pertussis vaccine.

Notes: The DTP1–DTP3 dropout rate was defined as the proportion of children immunized with the first diphtheria, tetanus and pertussis vaccine dose (DTP1) who failed to get the third dose (DTP3). District dropout rates for 2018 in each country were derived from data on vaccine coverage collected through the Joint Reporting Process of the World Health Organization (WHO) and the United Nations Children’s Fund (UNICEF). Districts in each country were divided into quintiles according to their DTP1–DTP3 dropout rate, with quintile 5 containing the 20% of districts with the lowest rates and quintile 1 containing the 20% with the highest rates. The difference between weighted average rates in quintiles 1 and 5 represents subnational inequality. National DTP1 and DTP3 immunization coverage rates were based on WHO/UNICEF national immunization coverage estimates.

Countries with the largest subnational inequalities in dropout rates also tended to be among those with the lowest DTP1 and DTP3 coverage, as derived from WHO/UNICEF national immunization coverage estimates (Fig. 5), such as Angola, the Central African Republic and South Sudan. In these countries, low DTP1 coverage combined with a high dropout rate resulted in estimated DTP3 coverage rates below 60%. Despite this general trend, the situation varied across countries, reflecting differences in immunization programmes. For example, whereas the Central African Republic and Nigeria both had relatively low DTP1 coverage (69% and 70%, respectively), the absolute difference in subnational dropout rates between quintiles 1 and 5 was twice as high in the Central African Republic as in Nigeria: 29.8 and 12.9 percentage points, respectively. 

Table 4 shows the potential improvements in national DTP1–DTP3 dropout rates that would be possible if subnational inequality were reduced or eliminated. In South Sudan, for instance, if the dropout rate in quintiles with a rate greater than the national average (i.e. quintiles 1 to 3) became equal to the national average, the national average dropout rate would decrease from 22.6 to 16.8%. Moreover, if the national average dropout rate equalled the rate in quintile 5 (i.e. 8.4%), the national rate would decrease by 14.2 percentage points. In all but three countries, the 2018 national dropout rate would be at least halved if subnational inequality were eliminated. Moreover, in six countries, the national dropout rate could potentially decrease by more than 70%: Benin, Burkina Faso, Cameroon, Côte d’Ivoire, Ethiopia and Madagascar. Across all 24 countries, DTP3 coverage could improve by 2.3 to 10.3 percentage points if there was no subnational inequality and dropout rates in all quintiles equalled the rate in quintile 5. For instance, in South Sudan DTP3 coverage would increase by 10.3 percentage points (from 56.2% currently to 66.5%) if there was no subnational inequality.

**Table 4:** Potential effect of reducing or eliminating subnational inequality in diphtheria–tetanus–pertussis immunization dropout rate on vaccination coverage, African Region, 2018

Country	Situation in 2018		Subnational inequality reduced^a^			Subnational inequality eliminated^b^	
	National DTP1–DTP3 dropout rate, %^c,d^		Estimated national DTP1–DTP3 dropout rate, %	Estimated improvement in DTP3 coverage, % points		Estimated national DTP1–DTP3 dropout rate, %	Estimated improvement in DTP3 coverage, % points
Angola	13.4		9.8	3.8		3.8	9.3
Benin	7.0		5.0	2.3		1.3	6.5
Botswana	9.2		6.7	2.1		4.2	4.4
Burkina Faso	3.5		2.3	1.3		0.6	3.2
Burundi	7.3		6.3	1.0		3.9	3.4
Cameroon	7.5		5.6	1.7		1.7	5.0
Central African Republic	22.1		19.1	2.8		13.0	8.6
Chad	10.8		8.5	2.0		4.6	5.4
Côte d'Ivoire	4.8		2.9	2.0		1.0	4.0
Democratic Republic of the Congo	6.0		4.8	1.2		2.6	3.4
Ethiopia	7.3		5.1	2.3		1.8	5.7
Guinea	7.4		5.5	2.1		2.7	5.1
Kenya	4.0		2.8	1.0		1.3	2.3
Madagascar	6.2		4.0	2.1		1.7	4.4
Malawi	4.2		3.3	0.9		1.4	2.7
Mali	15.8		13.8	2.3		8.4	8.4
Mauritania	13.3		10.2	3.0		3.7	9.3
Mozambique	4.9		3.1	2.2		2.1	3.4
Niger	5.7		4.5	1.3		2.2	3.7
Nigeria	7.9		6.0	2.0		3.1	4.9
South Sudan	22.6		16.8	4.2		8.4	10.3
Togo	4.9		3.6	1.2		1.9	2.8
Uganda	7.4		5.2	2.2		2.6	4.9
Zimbabwe	5.4		4.1	1.3		1.7	3.6

DTP1: first dose of the combined diphtheria, tetanus and pertussis vaccine; DTP3: third dose of the combined diphtheria, tetanus and pertussis vaccine.

^a^ Reduction in subnational inequality in the diphtheria, tetanus and pertussis immunization dropout rate between the first and third vaccine doses was defined as making the dropout rates in district quintiles with rates higher than the national average equal to the national average rate.

^b^ Elimination of subnational inequality in the DTP immunization dropout rate between the first and third vaccine doses was defined as making the dropout rates in all district quintiles equal to the rate in quintile 5 (i.e. the quintile with the lowest rate).

^c^ The DTP1–DTP3 dropout rate was defined as the proportion of children immunized with the first DTP vaccine dose (DTP1) who failed to get the third dose (DTP3).

^d^ National dropout rates were derived from data for 2018 on coverage of the first and third DTP vaccine doses collected through the Joint Reporting Process of the World Health Organization and the United Nations Children’s Fund.^24^

## Discussion

Our findings illustrate that national immunization dropout estimates can mask subnational pockets of low vaccine coverage where children are at risk of preventable disease and death. In particular, we found that the quintile of districts with the highest dropout rate tended to lag disproportionately behind the others and warranted extra targeted attention. Reducing the dropout rate in these districts would substantially improve national average DTP3 coverage. In certain regions of the world, the dropout rate goal has been set at 5% or less to emphasize the importance of completing vaccination series.^32^

An understanding of geographical variations in the DTP1–DTP3 dropout rate can help countries improve access to, and the utilization of, child health services, especially because the children concerned have accessed immunization services at least once to receive their first vaccine dose. Moreover, comparing subnational inequalities in the DTP1–DTP3 dropout rate with national DTP1 coverage provides an insight into the performance of the vaccine delivery system. For example, countries with low DTP1 coverage and relatively low subnational inequality in DTP1–DTP3 dropout rates, such as Benin and the Democratic Republic of Congo (Fig. 5), stand to benefit most from universal interventions aimed at increasing access to routine immunization. In these scenarios, substantial strengthening across the health system is required as many children are not even receiving the initial routine dose. In contrast, in countries with higher DTP1 coverage but marked subnational inequalities in DTP1–DTP3 dropout rates (e.g. Cameroon, Ethiopia and Mauritania), targeting districts where children are being left behind may be more effective in improving DTP3 coverage.

Using the dropout rate to monitor subnational inequalities is particularly useful in countries with high DTP1 coverage because the number of children who have not received any vaccine is low. However, more context-specific information may be required to determine the reasons why certain districts have higher dropout rates before appropriate solutions can be developed. In countries with low DTP1 coverage and high subnational inequality in DTP1–DTP3 dropout rates (e.g. Angola, Central African Republic and South Sudan), there are clearly systemic problems with both access to, and the utilization of, child health services. Such countries may benefit more by focusing on improving their health-system infrastructure.

Though it is well known that there are geographical gaps in immunization coverage within countries, few data may be available for systematically monitoring these gaps. Although administrative data can provide valuable, granular information on immunization coverage across the health system, for over 20 years immunization programme organizers have been concerned about limitations in data quality and the underlying reasons. The topic was first discussed by WHO’s Strategic Advisory Group of Experts (SAGE) in 1998,^33^ then again in 2011 and 2019.^34^^,^^35^ In October 2019, a SAGE working group presented an extensive report on immunization and surveillance data quality and use.^21^^,^^36^

As denominators, particularly at the local level, were known to be problematic, in our analysis we focused on numerators to minimize limitations associated with administrative data.^22^^,^^37^ Data quality issues related to numerators include: (i) incomplete reporting (e.g. not transcribing notes of doses given during outreach activities or not obtaining data on doses administered by private providers); (ii) errors in data recording (e.g. mistakenly marking a dose as the first, second or third dose based on the child’s age at vaccination rather than on the actual dose received); (iii) errors in data aggregation (aggregation is often done manually, usually at the end of each month); and (iv) implicitly assuming that children receive all their doses at the same location, whereas this is not always the case.^22^^,^^38^^,^^39^ These issues underscore the need for continued strengthening of health information systems to improve the quality of the vast amount of administrative data available. Multipronged interventions that focus on the local level where data are generated and increased use of data by individual health facilities and data aggregators have been shown to help improve data quality.^22^^,^^38^^,^^40^ A recent analysis suggested that data quality in the WHO African Region is improving (C Rau, University Medical Center Hamburg-Eppendorf, Germany, unpublished observations, 2021); in recent years fewer countries have had problems with inconsistent data that warrant further investigation than in the early years of the 21st century.

The method we adopted of grouping districts in each country into quintiles meant that comparisons of both within-country and between-country inequalities using summary measures were more robust because bias due to variations in the number of districts between countries was reduced. Nevertheless, the estimates produced in our analysis may not necessarily be representative of the situation in countries where a relatively high proportion of districts were excluded. Moreover, WHO/UNICEF national immunization coverage estimates of DTP1 coverage were based mainly on administrative data provided by individual countries and may have a high level of uncertainty.^41^

Subnational inequalities in immunization dropout are likely to exist in other African countries in addition to the 24 we investigated. Consequently, monitoring should be extended to more countries in the future. Although the dropout rate reflects only one aspect of immunization programmes, it is particularly useful for monitoring inequalities in areas where the reporting rate is sufficiently high and the number of children who receive no vaccine is low. However, this method should be combined with other indicators of immunization performance, such as data on vaccine coverage and on the proportion of children who receive no vaccine, which is not reflected in dropout rate. Monitoring geographical variations in immunization dropout can provide a basis for further investigations and, when conducted alongside the monitoring of other dimensions of inequality, can help generate a more comprehensive understanding of overall inequality.
